# Low-Cost AI-Enabled Optoelectronic Wearable for Gait and Breathing Monitoring: Design, Validation, and Applications

**DOI:** 10.3390/bios15090612

**Published:** 2025-09-16

**Authors:** Samilly Morau, Leandro Macedo, Eliton Morais, Rafael Menegardo, Jan Nedoma, Radek Martinek, Arnaldo Leal-Junior

**Affiliations:** 1 Postgraduate Program in Electrical Engineering, Federal University of Espírito Santo, Vitoria 29075-910, Brazil; samilly.morau@edu.ufes.br (S.M.); leandro.c.macedo@edu.ufes.br (L.M.); eliton.morais@edu.ufes.br (E.M.); rafael.menegardo@edu.ufes.br (R.M.); 2 Department of Telecommunications, Faculty of Electrical Engineering and Computer Science, VSB—Technical University of Ostrava, 17. listopadu 15, 70833 Ostrava, Czech Republic; jan.nedoma@vsb.cz; 3 Department of Cybernetics and Biomedical Engineering, Faculty of Electrical Engineering and Computer Science, VSB—Technical University of Ostrava, 17. listopadu 15, 70833 Ostrava, Czech Republic; radek.martinek@vsb.cz

**Keywords:** optoelectronic sensing, inertial measurement units, optical fiber sensors, gait analysis

## Abstract

This paper presents the development of an optoelectronic wearable sensor system for portable monitoring of the movement and physiological parameters of patients. The sensor system is based on a low-cost inertial measurement unit (IMU) and an optical fiber-integrated chest belt for breathing rate monitoring with wireless connection with a gateway connected to the cloud. The sensors also use artificial intelligence algorithms for clustering, classification, and regression of the data. Results show a root mean squared error (RMSE) between the reference data and the proposed breathing rate sensor of 0.6 BPM, whereas RMSEs of 0.037 m/s^2^ and 0.27 °/s are obtained for the acceleration and angular velocity analysis, respectively. For the sensor validation under different movement analysis protocols, the balance and Timed up and Go (TUG) tests performed with 12 subjects demonstrate the feasibility of the proposed device for biomechanical and physical therapy protocols’ automatization and assessment. The balance tests were performed in two different conditions, with a wider and narrower base, whereas the TUG tests were made with the combination of cognitive and motor tests. The results demonstrate the influence of the change of base on the balance analysis as well as the dual task effect on the scores during the TUG testing, where the combination between motor and cognitive tests lead to smaller scores on the TUG tests due to the increase of complexity of the task. Therefore, the proposed approach results in a low-cost and fully automated sensor system that can be used in different protocols for physical rehabilitation.

## 1. Introduction

The aging process naturally causes changes in the body. In the case of older adults, it is common to observe a reduction in muscle mass, which decreases strength, as well as a lower bone density, which weakens the skeletal structure [1]. These aspects affect posture, gait, and balance, factors that can increase the likelihood of falls [2]. In general, a fall can be defined as an unintentional event that results in the individual moving to a lower level than their initial position. This topic is very important in gerontology and is a major concern among researchers in the field, as it represents a serious public health issue around the world [3]. Falls can have serious physical and psychological consequences, including injuries, hospitalizations, impaired mobility, fear of falling again, restriction of activity, functional decline, institutionalization, and even death [4]. In the past 20 years, fractures resulting from falls have caused approximately 70% of accidental deaths in people over the age of 75 years. Older adults experience ten times more hospitalizations, resulting in higher government costs, and are eight times more likely to die from falls [5].

Identifying risk factors and implementing fall prevention strategies in the elderly is crucial to achieve effective healthcare interventions [6]. Functional movement assessments such as Timed Up and Go (TUG), Six-Minute Walk Test (6MWT), and balance tests are commonly used to assess risks such as falls and fatigue [7]. However, in both public and private healthcare settings, these assessments are often performed manually, which limits accuracy due to their reliance on subjective, experience-based evaluations [8]. This results in primarily qualitative insights, especially in analyzing gait symmetry and kinematic parameters [9]. To address these limitations, intelligent systems that can automate and quantify movement analysis in a portable, scalable, and user-friendly way may enhance service quality and clinical outcomes. Such technologies can promote better adherence to treatment and increase patient trust—factors that are especially important in a rapidly aging population [10]. For this reason, there is a growing demand for sensor technologies that can capture quantifiable data and deliver more precise and dependable outcomes. Given that older adults often face increased physical and motor limitations, their risk of experiencing domestic accidents also rises. Statistics indicate that approximately 35% of individuals over the age of 65 suffer at least one fall annually, potentially resulting in serious injuries and hospitalizations, which impose a significant burden on public healthcare systems [11]. In light of these concerns, wearable sensor technologies offer a valuable solution for enhancing the quality of life among the elderly. By enabling remote monitoring of physiological signals, these devices can help mitigate the effects and long-term consequences of falls [12,13]. A key benefit of wearable systems is their capacity to gather data outside traditional laboratory or clinical settings [14], making them suitable for applications such as fall detection and the monitoring of vital signs—including blood pressure, heart rate, and oxygen saturation [13,15]. In this context, the Internet of Things (IoT) emerges as a promising approach to ensure continuous, objective, and comprehensive monitoring. This not only reduces the reliance on human caregivers but also supports timely clinical decisions by identifying physiological changes and minimizing associated risks [16].

Considering the widely available sensor systems, recent research efforts have introduced various devices and methodologies for gait analysis using inertial measurement units (IMUs) [17], which can be used in combination with additional sensors [18]. IMU-based wearable systems offer a portable solution for the evaluation of human motion, but concerns about their reliability and consistency—especially in clinical settings—remain [19]. Common issues include orientation errors stemming from magnetometer use and drift caused by integrating gyroscope data over time. As a result, alternative methods that exclude magnetometer inputs have also been developed, which also include the use of artificial intelligence (AI) algorithms to automatically compensate for these errors and provide the regression or classification of different parameters during the gait analysis [20].

As another type of sensor, optical fiber sensors have gained attention as a viable option for physiological monitoring due to their inherent resistance to electromagnetic interference [21]. They also offer several practical benefits, including a compact and lightweight form factor, chemical durability, and the ability to support multiplexed signal transmission [22]. These features have enabled their successful integration into various systems [23,24,25], which also include advances in smart textiles with the incorporation of optical fibers to track various physiological metrics, including oxygen saturation [26], heart rate (HR) [27], breathing rate (BR) [28], and body temperature [29]. Typically, optical fiber-based HR and BR sensors are utilized in settings where patient movement is minimal, such as during sleep or within MRI environments [30]. Although these applications benefit from the technology’s electromagnetic immunity and compactness, sensor accuracy tends to decline under conditions involving significant motion. However, such an issue can be mitigated using different signal processing techniques that can be readily integrated in the sensor system [31].

Considering the previously published reports in optoelectronic wearable sensor systems, sensor systems are generally based on single-joint and single-plane measurement using optical fibers or in multiple planes using IMUs [18,32]. However, there are no reports (to the best of the authors’ knowledge) of simultaneous measurement of gait parameters and physiological responses using such optoelectronic systems. Moreover, the use of AI algorithms for the simultaneous assessment of physiological conditions and gait analysis leads to a novelty and important contribution in gait analysis, especially using low-cost optoelectronic systems with high accuracy, portability, and adaptability to different users.

**Figure 1:**
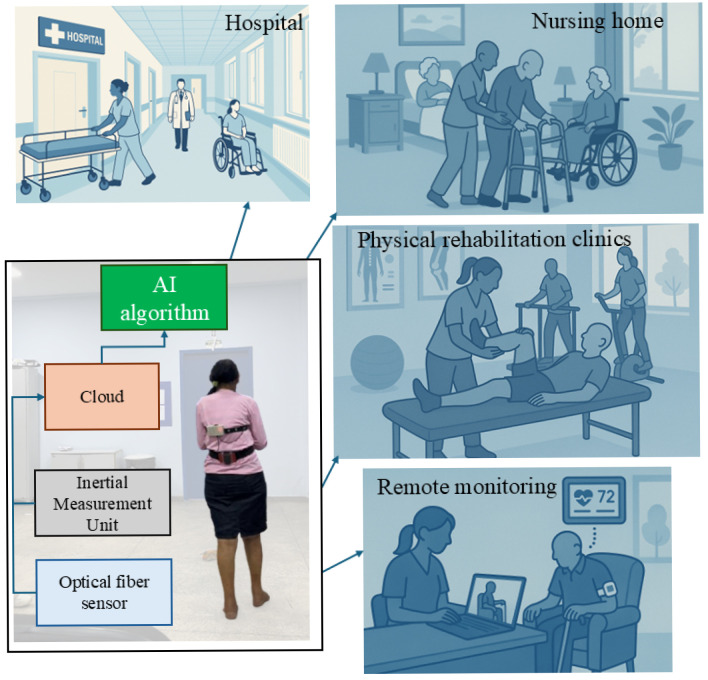
Schematic representation of the AI-enabled optoelectronic sensor system for gait analysis.

Taking into account this background, intelligent textiles and optical fiber biosensors are being integrated into conventional wearable devices for real-time monitoring of movement and physiological parameters. When combined with AI and cloud computing, these technologies support accurate, secure, and intelligent healthcare solutions [33,34,35]. The use of AI algorithms can provide a new tool for early fall risk detection outside clinical environments with the possibility of remote health monitoring, where the protocols of physical rehabilitation can be automated and are no longer restricted only to manual measurements, which can provide optimization of the treatments [20]. For these reasons, this paper presents the development of an optoelectronic wearable sensor system for portable monitoring of the movement and physiological parameters of patients. The proposed approach uses AI algorithms for clustering, classification, and regression of the data, which provide important tools for the clinicians and can be used not only in clinical environments but also in remote health monitoring conditions. Figure 1 presents a summary of the proposed solution with not only the proposed application in TUG, but also the potential applications in home monitoring, physical rehabilitation clinics, hospitals, and nursing homes.

## 2. Materials and Methods

The proposed optoelectronic approach is based on two integrated systems: (i) an optical fiber-integrated chest belt and (ii) a belt-integrated IMU. In this case, the sensor systems are used in conjunction for the biomechanical assessment of the patients. In this case, the optical fiber sensor is employed for heart rate and breathing rate estimation, whereas the IMU is placed on the lumbar region of the patient for the biomechanical assessment, which includes not only the angles of the pelvis, but also the spatiotemporal parameters of the gait, including the time taken for each phase of the experiment, leading to the estimation of a patient’s fall risk.

For low-cost IMU development, a 3-axis accelerometer and 3-axis gyroscope are used to measure the kinematic parameters during the patient’s gait. These sensors not only enable the measurement of accelerations and angular velocities in all directions, but also the assessment of Euler angles and linear displacements, which can completely describe the gait of the patient. These data are used to provide a quantitative analysis of the patient’s performance in each automated physical therapy protocol in the proposed solution. The data are then transmitted via Bluetooth. It is important to mention that the microcontroller of the device is an ESP32 board with integrated Bluetooth connection, which is used to transmit the data to a local gateway. All components are powered by batteries, enabling the wireless operation of the device.

**Figure 2:**
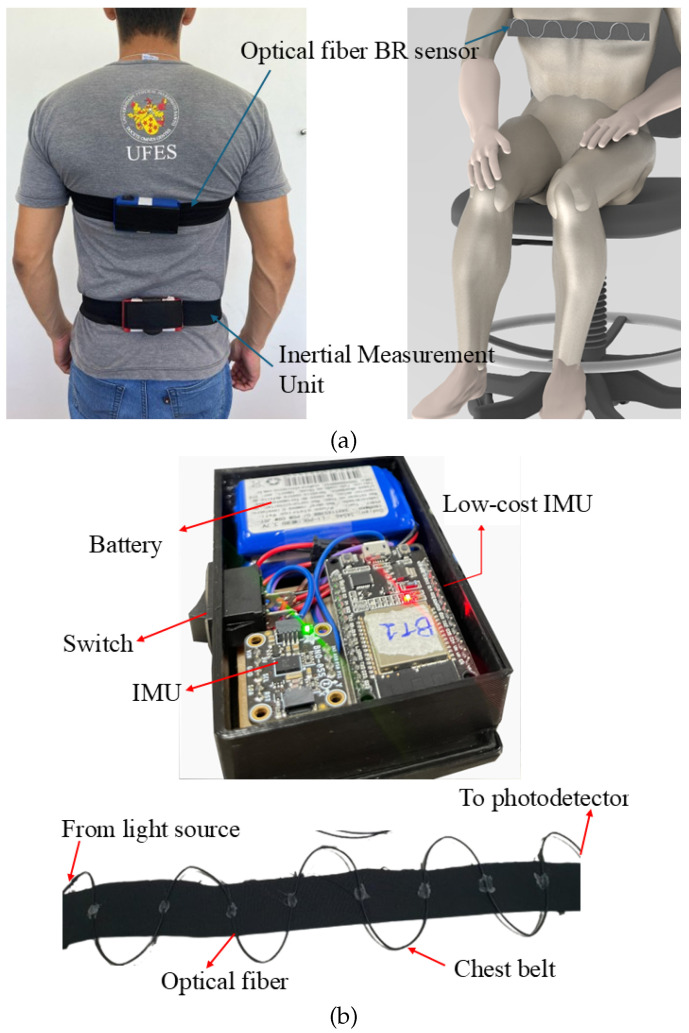
(**a**) Overview of the proposed optoelectronic sensor system for gait analysis. (**b**) Photographs of the components used in the sensor system.

For the optical fiber-based breathing rate sensor, the intensity variation principle is employed. In this case, the proposed sensor detects curvature changes caused by breathing through a polymer optical fiber (POF) attached to a chest belt. To improve sensitivity, lateral sections are created by removing parts of the fiber’s cladding and core using controlled sandpaper [36], where 8 lateral sections are created along the optical fiber to increase its sensitivity as a function of the chest movement during respiration. Thus, the chest movements during breathing lead to a curvature on the optical fiber, which results in optical power variations along the fiber, where such periodic variations are analyzed using Fast Fourier Transform (FFT) to obtain the breathing rate from the peak of the frequency spectrum. In addition, the sensor operates in transmission mode using a 660 nm LED (IF-E97, Industrial Fiber Optics, Tempe, AZ, USA) as the light source and a phototransistor (IF-D92, Industrial Fiber Optics, Tempe, AZ, USA) to detect optical power variations. It is also important to mention that the POF used is a polymethyl methacrylate (PMMA) POF (HFBR-EUS100Z, Industrial Fiber Optics, Tempe, AZ, USA) with a 980 µm core, 20 µm thick fluorinated polymer cladding, and a polyethylene coating. Figure 2a shows the setup of the proposed optoelectronic sensor system for automatic gait analysis positioned in a patient, whereas Figure 2b presents an overview of the components used in the sensor system.It is worth noting that the parameters of the lateral sections (such as length and depth) made on the optical fiber influence the linearity, sensitivity, and resolution of the sensor (as demonstrated in [36]). For this reason, the length and depth of the lateral sections are controlled at around 18 mm and 0.8 mm, respectively. In addition, the surface roughness of the sensor system is controlled using a controlled sandpaper with a grit size of 800 for the abrasive removal of material. Moreover, the excessive depth and length of the lateral section can harm the robustness of the system. Thus, the lateral section parameters provide high sensitivity and linearity (as demonstrated in [36]) and also result in long-term durability of the sensor system. This system can be reused for different subjects since it can be adapted to different body types. User comfort is assessed by user feedback, where all users described the system as comfortable enough to not harm their natural movement.

Before applying the sensors for biomechanical analysis, they undergo a characterization process. For the characterization of the breathing rate sensor, an experiment is conducted using a metronome to establish known periodic signals (related to breathing rates) and to compare them with the frequencies measured by the proposed sensor system. The metronome is set to five different frequencies (related to 10 BPM, 13 BPM, 15 BPM, 17 BPM, and 20 BPM) and the POF-embedded chest belt is manually expanded in sync with the metronome’s beats. Five tests are conducted at each metronome frequency to ensure repeatability and the mean values for each condition are analyzed. The sensor response is compared with the metronome, since controlled movement of the chest belt provides direct variation in sensor responses. In addition, comparing it with the commercial spirometer would harm its implementation in gait tests. Then, the low-cost IMU developed is compared with the commercial measurement system (BTS Baiobit, Milan, Italy). In this case, both sensors are applied in the same position on the user, who is asked to perform the TUG experiment to evaluate the responses in all 3 axes of the accelerometer and the gyroscopes of both devices.

For the first set of application tests, the bipedal support balance test involves the individual standing on both legs in a static position, with the feet parallel and close together, and it can be performed with the eyes open or closed. The parameters analyzed in this test include the oscillation of the Center of Pressure (CoP) and the postural stability index, with the goal of evaluating balance under stable conditions and the distribution of weight between the feet. In this case, the tests are performed with eyes opened, but with two different positions of the feet, where the first one is conducted with a wider distance between feet, the so-called wide base, whereas the second one is conducted with the feet close to each other, the so-called narrow base.

**Figure 3:**
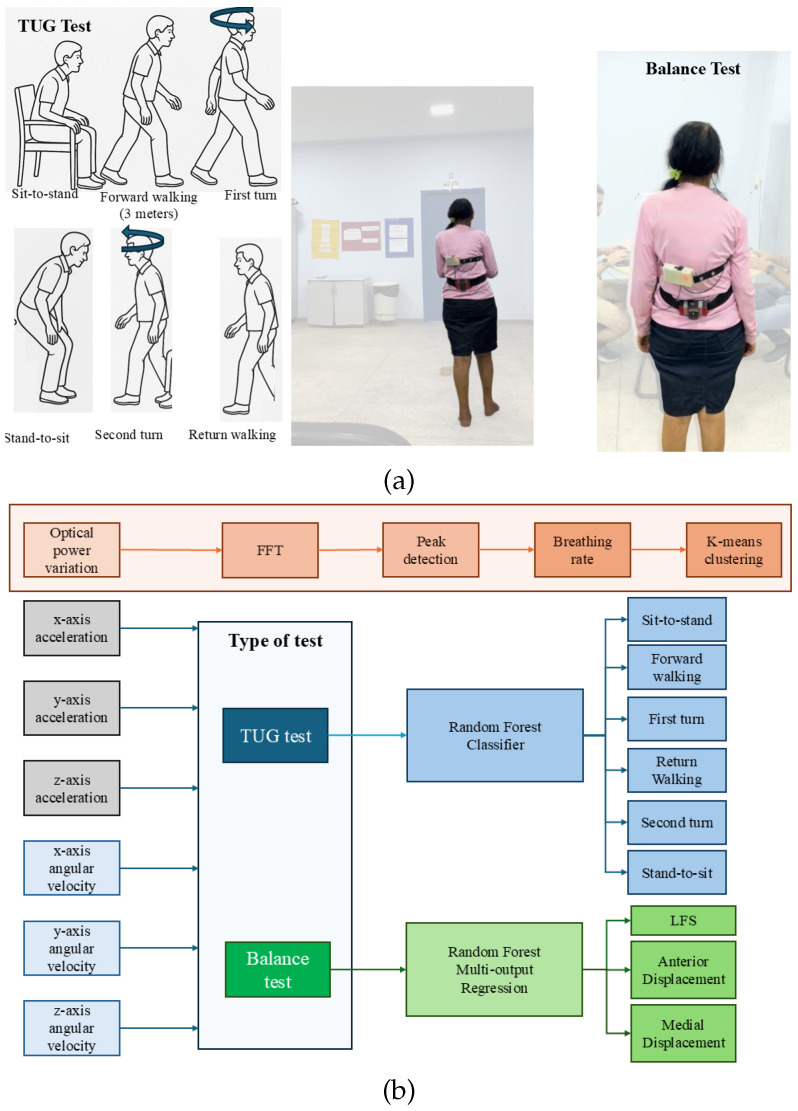
(**a**) Experimental setup for the validation tests. (**b**) Flowchart of the proposed AI algorithm for data analysis.

The TUG test was designed to evaluate mobility, balance, and fall risk in older adults. It involves measuring the time taken for a patient to walk a distance of three meters. In this study, a volunteer performed the TUG test in three variations: the TUG test alone (so-called conventional TUG), the TUG test combined with a cognitive task (recalling the number prior to the one mentioned by the evaluator while walking), and the TUG test combined with a motor task (juggling a ball while walking). For both validation tests, i.e., TUG and balance tests, the breathing rate estimation unit is placed on the chest of the patient, whereas the low-cost IMU is placed on the lower part of the lumbar spine (L4, L5 position) and secured to the patient using an elastic strap, as shown in Figure 3a.

After the sensor characterization and the definition of the tests, it is possible to implement the AI algorithms for the sensor data interpretation, classification, and additional analysis. In this context, the breathing rate sensor is analyzed in an unsupervised approach for the data clustering of the breathing rates of each patient. The goal is to verify abnormalities in the BR during the different test protocols, where such variations can be mostly related to fatigue during physical activities. In addition, the fatigue during the exercises can influence the biomechanical responses during tests, which can directly affect gait parameters. In this context, we employ the K-means clustering algorithm in conjunction with the silhouette score to estimate the optimum number of clusters, where the number of clusters is varied from 2 to 20 and the silhouette score is analyzed for each number of clusters. In this analysis, a data set with 145 samples (considering all subjects) is used for the clustering in which the optimal number of clusters is analyzed using 300 maximum interactions.

The other part of the AI algorithm includes the 3-axis accelerometer, 3-axis gyroscopes, and the time stamp responses as the inputs for a supervised approach for the assessment of the spatiotemporal parameters of the TUG tests, namely the times for sit-to-stand, forward walking, first turn, return walking, second turn, and stand-to-sit. Furthermore, the fall detection risk classification can be obtained from the sum of the detected intervals. It is important to mention that the reference values for the algorithm training and testing are obtained from the BTS Baiobit sensor, whereas the fall risk classification is performed according to conventional methods in the literature, which are based on the total time of execution of the conventional TUG. In this case, the conventional fall risk classification is as follows: (i) less than 10 s: low fall risk (generally indicates normal mobility); (ii) 10–19 s: moderate fall risk (indicates some instability or mild difficulties); (iii) more than 20 s: high fall risk (indicates a high risk of falls, may be associated with serious balance, coordination, or muscle strength issues). In this case, each of the aforementioned parameters of each phase of TUG is obtained through the Random Forest classifier in which the data from each phase are classified and the time interval for each phase is obtained from the time difference between the first and last classification of the phase. For example, if the sit-to-stand activity is first detected at 0.2 s (from the time stamp of the sensors) and last detected at 1.2 s, the interval for this activity is 1 s. For the training and testing of the algorithm, a 70/30 proportion was used, which is a common division with most of the data in the training process. In addition, k-fold cross-validation (k = 5) was used. Since the algorithm was trained considering all subjects, a data set with more than 90,000 samples was used in which 54 estimators and 6 maximum features were used. Figure 3b shows the flowchart of the algorithm for the proposed AI algorithms.

For the tests with patients, the sensors are positioned on the user’s chest (for the respiration rate) and the lumbar region (for the biomechanical analysis). The test protocols include the conventional TUG, the TUG with motor tasks, and the TUG with cognitive tasks, as well as the balance tests, which are conducted with wider and narrower positions of the feet. The patients are at least 65 years old and 12 patients are analyzed, 3 male and 9 female. Despite the differences in gender distribution and relatively small sample size, the proposed sensor system can be applied in different groups with almost no adjustment, since the sensor system can be adjusted to different body types and sizes. Furthermore, the proposed wearable device can be applied to different groups with special circumstances, since the sensors can be positioned in different regions on the body and can be applied to diverse populations, including obese groups or those with spinal deformities with only small changes occurring due to using longer straps for sensor positioning. Table 1 shows relevant data of the subjects, including the age, gender, mobility conditions, and clinical conditions. It is important to mention that we have received the informed consent of the patients for the tests, which were performed following the guidelines of the National Health Council with the protocols approved by the Research Ethics Committee through the National Commission in Research Ethics—CONEP (Certificate of Presentation for Ethical Appreciation—CAAE: 77121823.5.0000.5542).

## 3. Results and Discussion

The analyses of the results are performed in different steps, where the first step is to demonstrate the optoelectronic sensor system’s characterization. Thereafter, the balance tests are analyzed under two different conditions, that is, the wide and narrow position of the feet, and the oscillations of the sensors are analyzed under both conditions. This test is important not only for the assessment of typical variations in the accelerometer and gyroscope for each patient, but also the normal ranges of breathing rates for each patient, which are used as the basis to analyze the fatigued or abnormal breathing rates during the physical activities. Then, the analysis is performed only on the conventional TUG tests and their variants, namely the TUG with cognitive tasks and TUG with motor tasks. As the last step of the results analysis, a discussion and comparison between the protocols are also presented in terms of a statistical analysis.

### 3.1. Optoelectronic Sensor System Characterization

**Figure 4:**
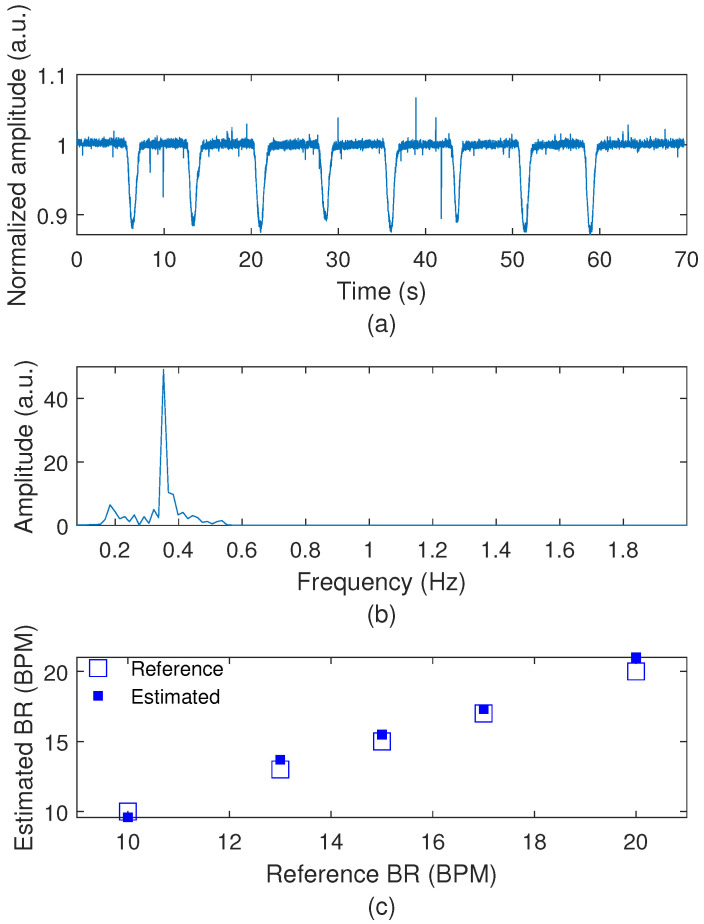
(**a**) Time-domain response of the proposed breathing rate sensor. (**b**) FFT of the sensor responses. (**c**) Breathing rates estimated with the proposed sensor system.

The characterization results for the breathing rate (BR) sensor system are presented in Figure 4, where it is possible to observe the results in time- and frequency-domains in Figure 4a,b, respectively. The time-domain results show a periodic signal with similar amplitude (mean amplitude variation of 0.9 a.u.), whereas Figure 4b shows the FFT of the signal in which there is a peak frequency at 0.34 Hz (resulting in around 20 BPM), which is the frequency used for the sensor characterization. Then, the results in Figure 4c demonstrate the estimated and reference frequencies applied on the optical fiber breathing sensor, where it is possible to verify the high accuracy of the proposed sensor, where a root mean squared error of 0.6 BPM is obtained.

**Figure 5:**
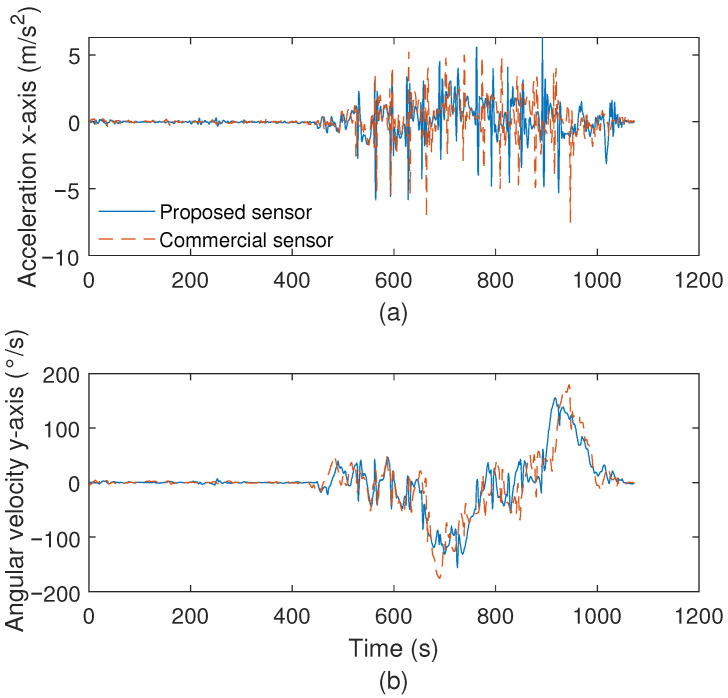
(**a**) Comparison between the proposed and commercial IMUs for the accelerometer on the x-axis. (**b**) Comparison between the proposed and commercial IMUs for the angular velocity on the y-axis.

For IMU characterization tests, the results are presented in a TUG test with a healthy individual using both sensors at the same time. The results presented in Figure 5a show the accelerometer results in the x-axis, whereas Figure 5b presents the angular velocity responses in the y-axis. It is important to mention that these results were chosen to provide a better visualization of the comparison between sensors. However, a comparison between the angular velocities and accelerations for all axes is presented in Table 2, where it is possible to verify the mean errors between the proposed low-cost IMU and the commercial system. The results in Figure 5 show a high correlation between both IMUs, in which the errors of 0.06 m/s^2^ and 0.2456 °/s are obtained for the accelerometer on the x-axis and angular velocity on the y-axis, respectively. If all axes and parameters are compared (see Table 2), the high correlation between both approaches is also confirmed, where the mean and standard deviation of errors for all conditions analyzed are around 0.037 m/s^2^ and 0.27 °/s for the acceleration and angular velocity, respectively, which can be considered as small errors between sensors.

**Figure 6:**
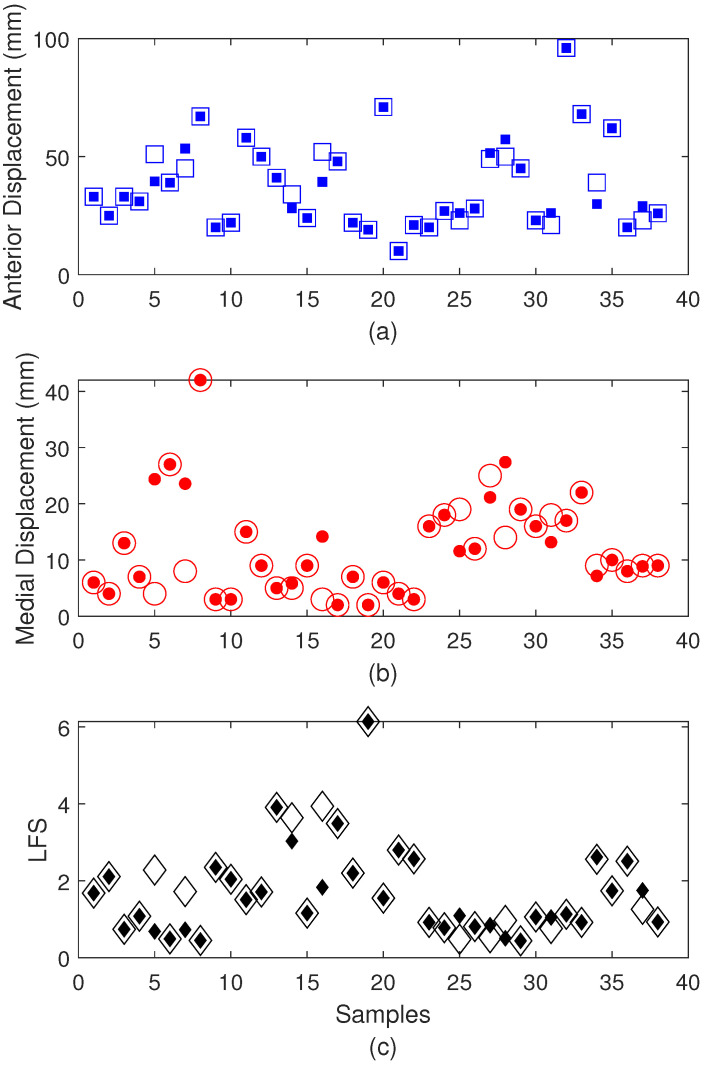
Estimated and reference values for the balance testing after the AI algorithm’s application for (**a**) open squares: reference anterior displacement; filled squares: estimated anterior displacement, (**b**) open circles: reference medial displacement; filled circles: estimated medial displacement, and (**c**) open diamond: reference LFS; filled diamond: estimated LFS.

Thereafter, the sensor characterizations also include the AI algorithm for feature extraction for each test protocol. In this case, the IMU is analyzed for different test protocols, where the first step is to choose the protocol (between balance and TUG). For the balance tests, a multi-output regression Random Forest algorithm is employed using the angular velocities and accelerations and x-, y-, and z-axes as the inputs, whereas the outputs are the anterior and medial oscillations and Local Fractal Stability (LFS), which are common indices for postural stability testing. The results in Figure 6 show the reference and estimated values (using the proposed optoelectronic sensor system) for the anterior and medial oscillations as well as the LFS in Figure 6a–c, respectively. The results indicate high accuracy for the parameter detection, where the root mean squared error (RMSE) for medial and anterior displacements are around 5.0 mm and 4.0 mm, respectively. Furthermore, the LFS estimation results in an RMSE of 0.49 after the AI algorithm’s application.

**Figure 7:**
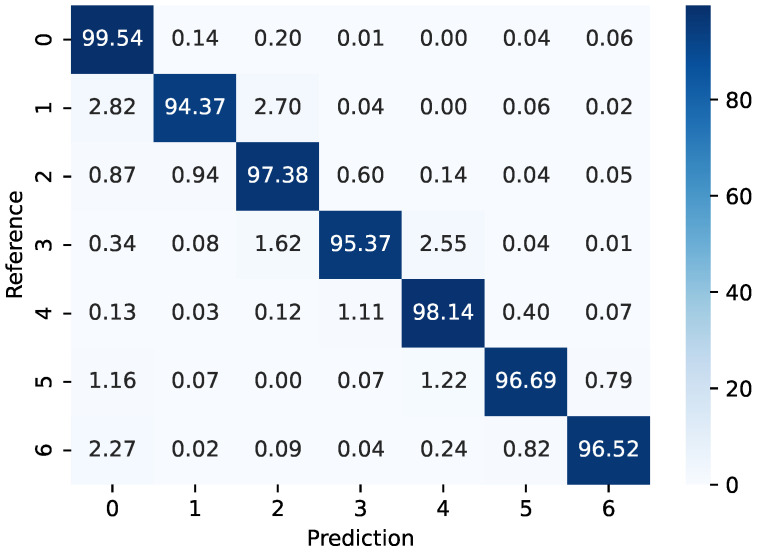
Confusion matrix for the class prediction using the proposed AI-enabled device.

For the TUG protocol, the multi-output Random Forest classifier uses the angular velocities and accelerations and x-, y-, and z-axes as the inputs. However, the outputs in this case are the time for sit-to-stand, time for walking forward, turn time for return walking, and time to sit down. For each interval, the maximum acceleration is analyzed. In addition, the total time of the test is obtained from the sum of each interval, where the classification of the fall risk is made according the total time for the test, since this is the conventional method for such a classification. For the classification analysis, Figure 7 shows the confusion matrix of the reference and estimated times for the TUG tests, where accuracy above 95% is obtained for each class. In addition, the classes are numbered from 0 to 6, where each class is obtained considering each phase of the TUG test. In this case, class 0 refers to the start of the test, whereas classes 1 and 2 refer to the stand-to-sit and walking forward phases, respectively. Then, the first turn is considered as class 3 and walking back to the chair is considered class 4. Thereafter, there is a second turn when the patient reaches the chair, which is considered class 5. Finally, class 6 refers to the stand-to-sit condition in which the test is finished.

The results in Figure 7 indicate the feasibility of the proposed sensor system, where a high accuracy between the reference and estimated signals is obtained in all analyzed cases. It is important to mention that the times of each phase of the TUG tests are obtained from the time stamp at which each class is classified and the intervals for each phase during the TUG test are estimated from the time difference between the first and last times at which each activity is classified.

**Figure 8:**
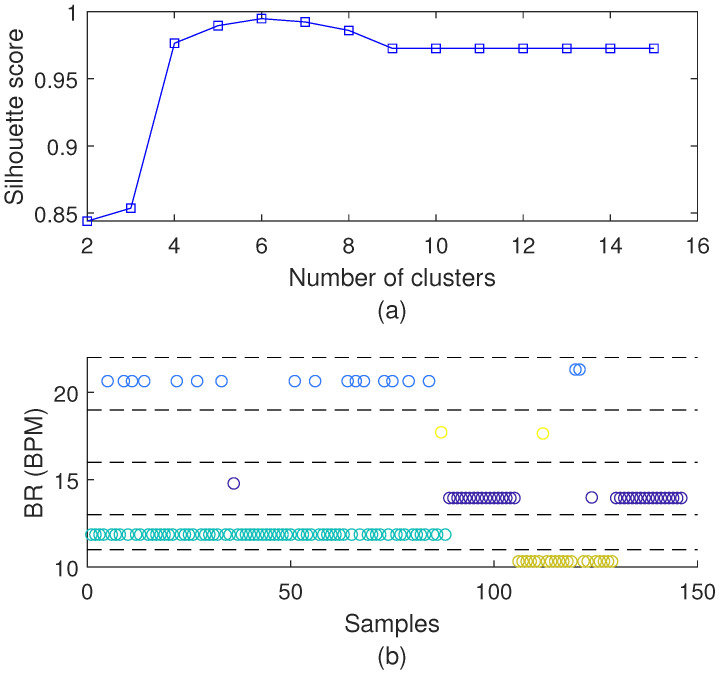
(**a**) Optimal number of clusters and (**b**) clustering of the breathing rate sensor systems at different respiration frequency conditions, blue circle: class 1; purple circle: class 2; cyan circle: class 3; yellow circle: class 4.

Finally, for the analysis of the breathing rate, we use the unsupervised algorithm to group the measured breathing rates. The results are obtained from the additional tests at different respiration frequencies ranging from Bradypnea (below 12 BPM, but above 0 BPM) and normal breathing (between 12 and 20 BPM) to Tachypnea (above 20 BPM). Figure 8 shows the clustering of the breath sensor system, where the optimal number of clusters is five, which is related to one of the peaks of the silhouette score presented in Figure 8a. This approach is used for the breathing rate clustering during the balance and TUG tests, where the results for the characterization tests are presented in Figure 8b, in which it is possible to verify five different regions of the BR and such regions will be used for the analysis of the correlation between the breathing rates and the performance parameters during TUG and balance tests.

### 3.2. Balance Test Results

**Figure 9:**
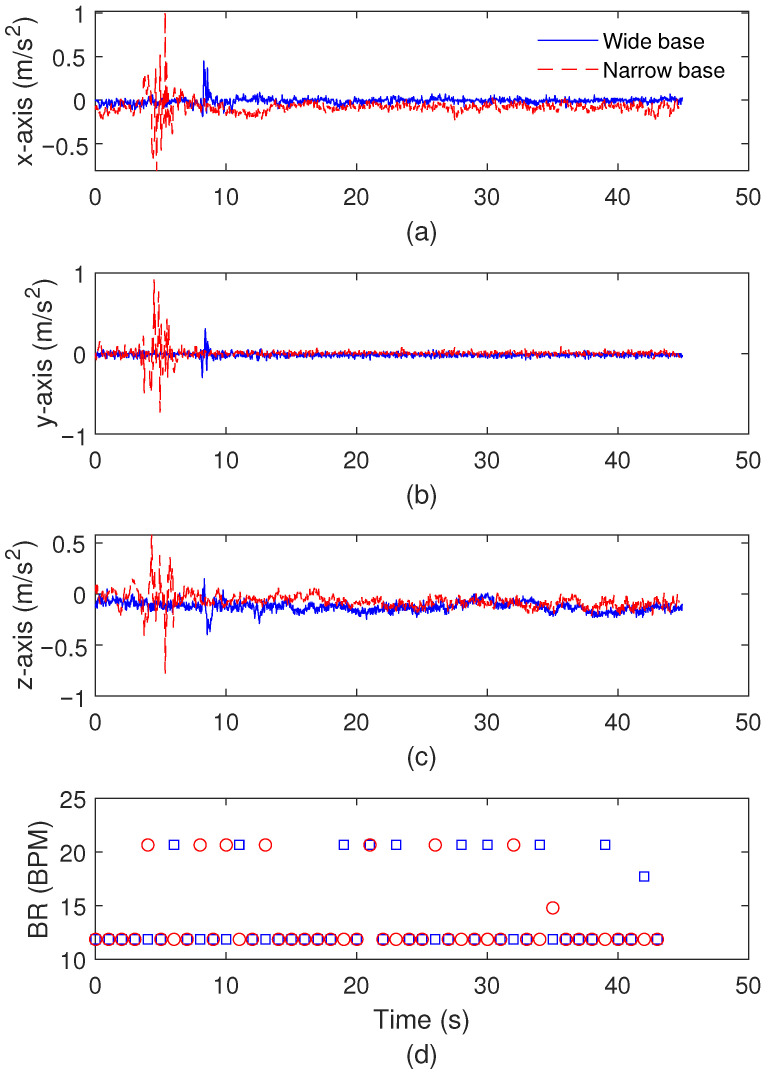
Acceleration variation of (**a**) x-axis, (**b**) y-axis, and (**c**) z-axis. (**d**) Breathing rate variation throughout the test.

The first application test is the balance test in which the patient stays at the same position with eyes open in two different conditions: (i) a wider distance between the feet and (ii) a narrower distance between the feet. The results for the biomechanical analysis are presented in Figure 9 for one test at each condition, where it is possible to analyze the signal variations along the test with wider and narrower positions of the feet. In this case, the accelerations at x-, y-, and z-axes are presented in Figure 9a–c, respectively, where it is possible to verify small variations in the accelerations in the test. In this context, the maximum accelerations for the x-, y-, and z-axes in the wider base tests are around 0.45 m/s^2^, 0.31 m/s^2^, and 0.40 m/s^2^, respectively. Similarly, the maximum accelerations for x-, y-, and z-axes in narrower base tests are around 1.02 m/s^2^, 0.92 m/s^2^, and 0.58 m/s^2^, respectively. In addition, the results in Figure 9d present the breathing rate variation throughout the test, where there are mean and standard deviations of 13.79±3.62 BPM and 13.32±3.25 BPM along the tests of wide and narrower bases, respectively. Such results indicate the high stability of the patient and can also be used to set the normal variations in the analyzed parameters prior to the TUG test’s implementation.

Table 3 presents an overview of the parameters analyzed in the balance tests, where conditions with wider and narrower feet positions are also compared. In this case, the mean LFS, accelerations at different axes, and displacement of anterior and medial planes are presented for both balance testing conditions. The results indicate higher variations in the displacements and angles when there is a narrower position of the feet, which is expected, since it is a condition with less stability. However, there are no significant variations in the breathing rates considering both conditions. Thus, the variations when both conditions are compared are related to the biomechanical parameters, especially the LFS, which presents a 45.2% reduction when the narrower position is compared to the wider foot positioning condition.

### 3.3. TUG Test Results

**Figure 10:**
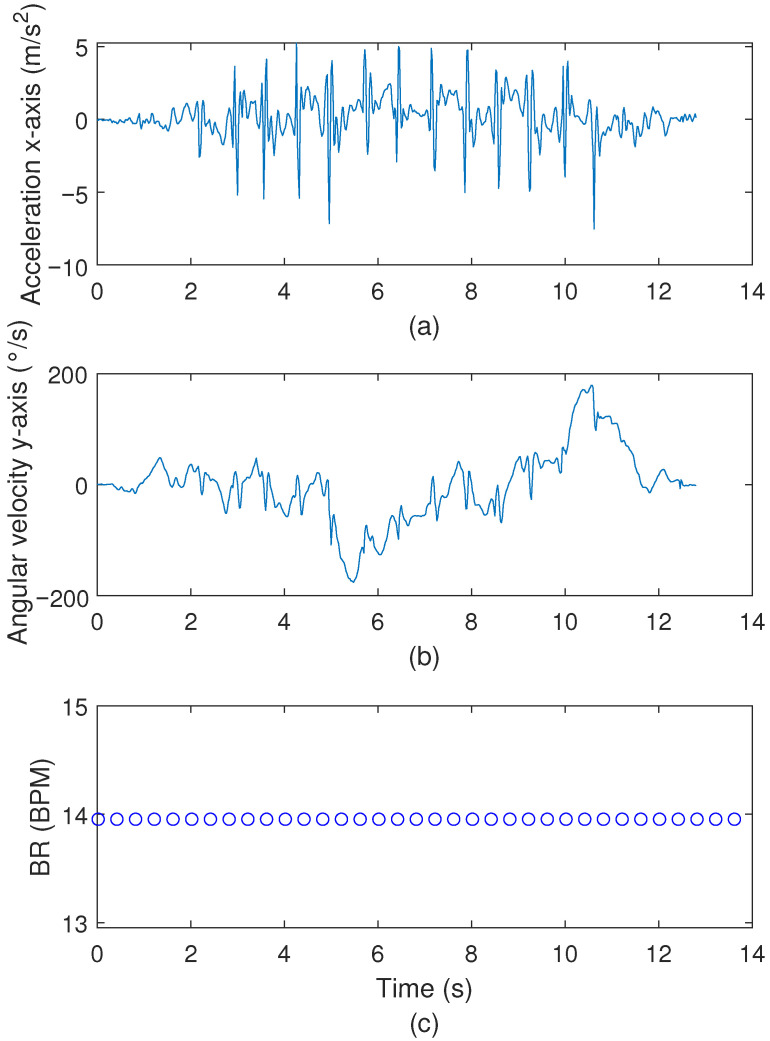
(**a**) IMU accelerometer response for the x-axis of the conventional TUG test. (**b**) IMU angular velocity response for the y-axis of the conventional TUG test. (**c**) Measured breathing rates during the conventional TUG test, blue circle: estimated BPM.

The sensor responses for the conventional TUG are presented in Figure Z, where Figure 10a shows the accelerometer at the x-axis, Figure 10b depicts the angular velocity at the z-axis, and Figure 10c presents the breathing rate measured throughout the test. It is worth noting that the sensor responses show the typical instants for each event during the conventional TUG (namely, sit-to-stand, forward walking, first turn, backwards walking, second turn, and stand-to-sit). The indication of the start and end of each event can be obtained from the results in Figure 10a,b. In this experiment, the patient was able to complete the task in 10.96 s, where the times of 1.26 s, 5.8 s, and 9.24 s were found for the sit-to-stand, first turn, and stand-to-sit events, respectively. Considering Figure 10c, it is possible to verify a negligible variation in the breathing rate, which can be regarded as a small variation during the test.

**Figure 11:**
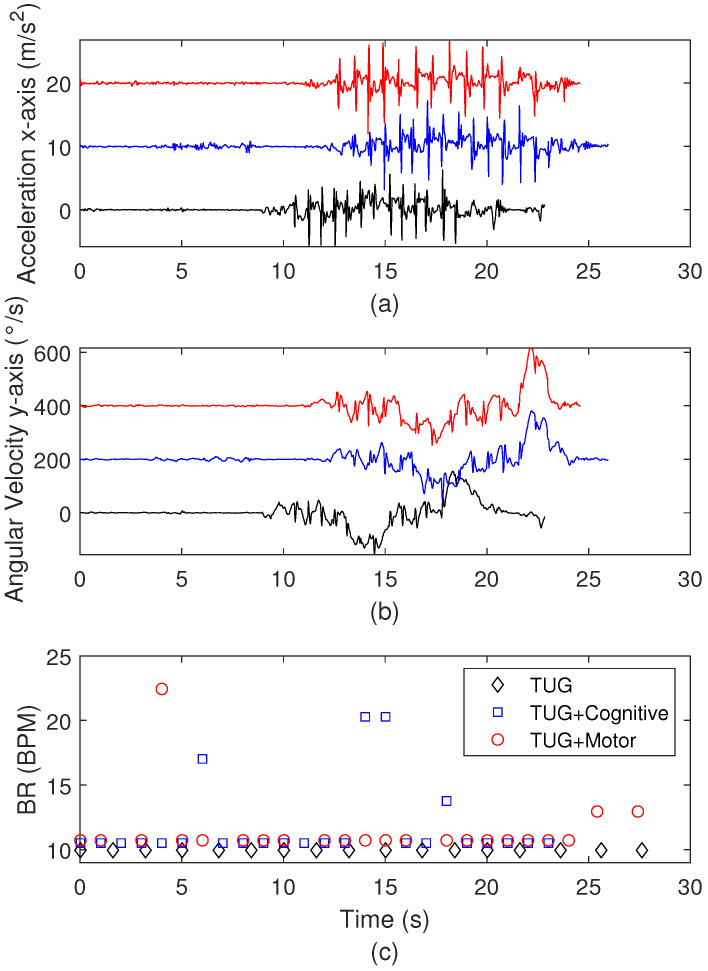
Comparison of conventional TUG, motor task TUG, and cognitive task TUG for (**a**) IMU accelerometer response for the x-axis with offsets of 10 m/s^2^ and 20 m/s^2^; (**b**) IMU angular velocity response for the y-axis with offsets of 200 °/s and 400 °/s; (**c**) measured breathing rates.

Then, to provide a comparison between the three different TUG approaches, Figure 11 shows the comparison between the results of the conventional TUG, motor task TUG, and cognitive task TUG. The comparisons are made as a function of the acceleration at the x-axis, angular velocity at the y-axis, and breathing rates in Figure 11a–c, respectively. The results demonstrate the influence of the motor and cognitive tasks on the gait tests, where it is possible to observe a higher variation in the signals during the TUG combined with motor activity, considering both movement parameters analyzed. In addition, the breathing rates at different conditions indicate a 30% variation when all tests are compared. In this case, the highest breathing rates are obtained from the TUG with cognitive activity, where it is possible to infer that the dual tasks, i.e., the gait combined with cognitive and motor tasks, can result in higher differences in the signals, which may be related to focus and fatigue during the tests.

In order to demonstrate the spatiotemporal parameters during gait (in conjunction with the breathing rates), Table 4 shows the summary of the results obtained in all TUG tests with one subject, where the results in the table also present the means and standard deviations of the acceleration and angular velocities in different axes. The Random Forest algorithm trained and discussed in Section 3.1 results in the spatiotemporal parameters of Table 4. The results indicate the influence of the dual task conditions on the biomechanical analysis, where there is a 7% increase in the total time for the test when the motor activity is analyzed in conjunction with the TUG, in which the motor activity resulted in highest variation in the time interval. Comparing the cognitive and motor tests, it is possible to verify that the motor test presented a 3% increase in the time, which indicates that the motor dual task can lead to a reduction in focus and increase in fatigue, where such parameters can be monitored in real time.

### 3.4. Discussion and Analysis

**Figure 12:**
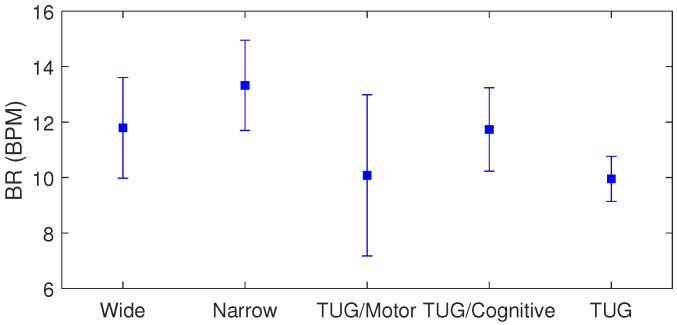
Mean and standard deviation of the breathing rates during different test protocols.

The statistical analysis of the data provides insights of the influence of each protocol on the biomechanical and physiological parameters as well as demonstrates the suitability of the proposed low-cost sensor system approach. The results of the breathing rates for all tests are presented in Figure 12. The results represent the results of all patients throughout the test. In this case, it is possible to verify the highest variation in the breathing rate for the TUG combined with the motor task. The mean values of the breathing rate are around 12.0 BPM, whereas the deviations of 1.6 BPM, 0.6 BPM, and 2.9 BPM were found for the balance tests with wide and narrow basse, conventional TUG, and TUG combined with motor tasks.

**Figure 13:**
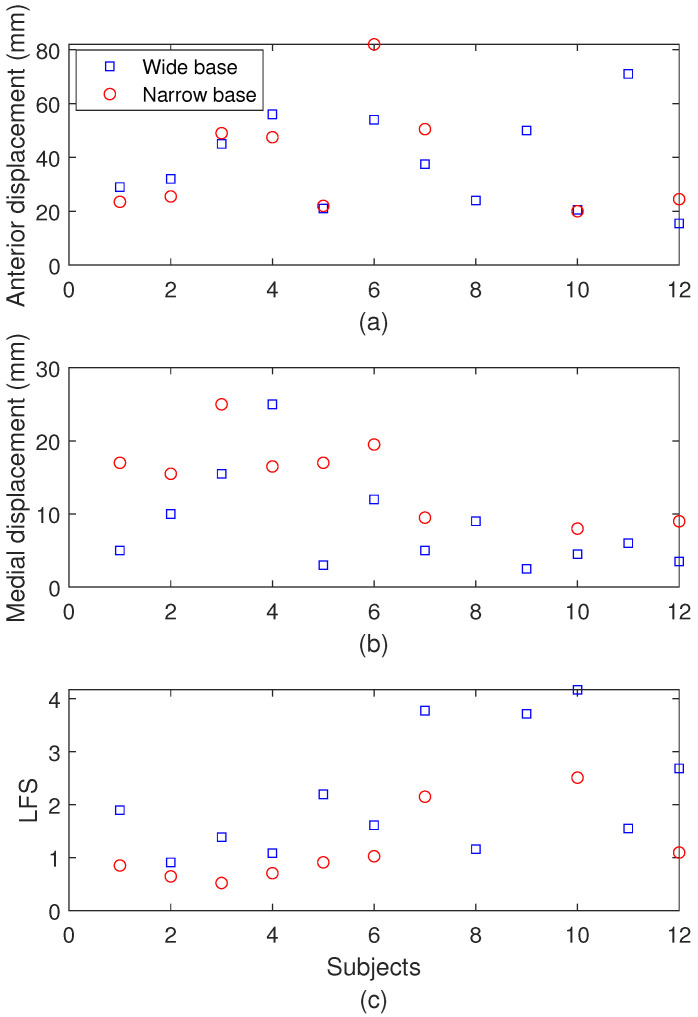
(**a**) Medial displacement for all patients during balance tests. (**b**) Anterior displacement for all patients during balance tests. (**c**) LFS for all patients during balance tests.

The biomechanical results of the balance protocol for each subject are presented in Figure 13, where the anterior displacement, the medial displacement, and the LFS are shown in Figure 13a–c, respectively. For the test with a wider base position, the results indicate the highest anterior displacement, medial displacement, and LFS for subjects 11, 4, and 10, respectively. It is also important to mention that the mean displacements with a wider base are smaller than the ones with a narrower base, which is expected since it is a condition with less stability. Thus, as higher LFS indicates higher postural control, the wider base tests present higher LFS values. In this context, there is an increase of 1% and almost 90% in anterior displacement and medial displacement, whereas there is an almost two-fold decrease in LFS when the mean results are compared for all patients in balance tests with wider and narrower bases. Considering the narrower base balance tests, subjects 6, 3, and 10 present higher values of anterior displacement, medial displacement, and LFS, respectively. The comparison with the mean values of the other subjects indicates an increase of 2.5 times in medial displacement for subject 6, whereas the increase in anterior displacement of subject 4 is around 10%. Similarly, the LFS increase in subject 10 is around 2.5 times greater when compared with the mean values of the other subjects.

**Figure 14:**
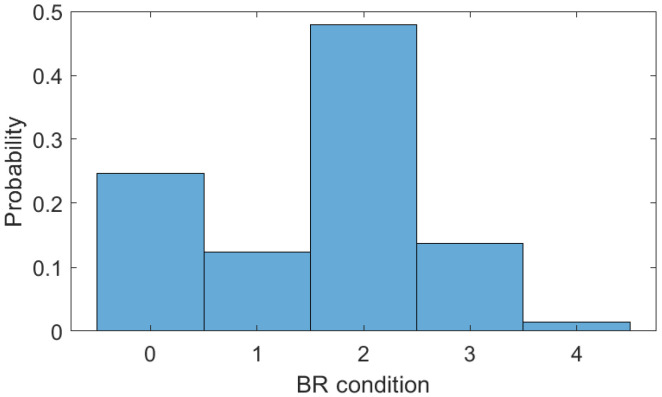
Breathing rate distribution for TUG test combined with motor task.

Then, for the TUG tests, the clustering of the breathing rates is employed, where the clustering conditions are analyzed, i.e., condition 0 (breathing rates below 10 BPM), condition 1 (between 11 BPM and 13 BPM), condition 2 (between 14 BPM and 16 BPM), condition 3 (between 17 BPM and 19 BPM), and condition 5 (above 19 BPM). Figure 14 shows the distribution of the different breathing rate conditions for each type of test, where it is possible to verify condition 2 (i.e., normal breathing condition) as the most common one. However, for the TUG with the motor task, the conditions of abnormal respiration are around 15%, which is related to the increase in difficulty of the TUG test when a dual task is incorporated.

**Figure 15:**
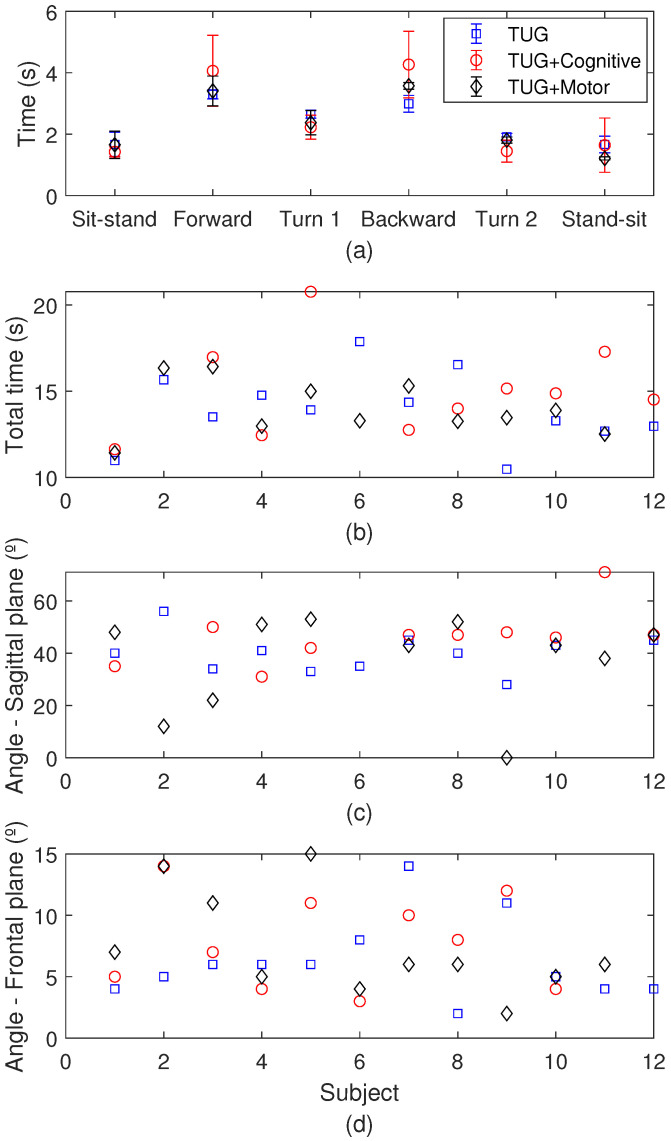
(**a**) Times for each phase of the TUG test. (**b**) Total times of TUG for each subject. (**c**) Maximum angles in the sagittal plane for the TUG test. (**d**) Maximum angles in the frontal plane for the TUG test.

In the biomechanical analysis, the results for each subject are presented in Figure 15a as function of the time to perform each phase of the TUG, where it is possible to verify the longest time taken to perform the return walking. Comparing the results for each subject in Figure 15b as a function of the total time, subject 6 presents the longest time to perform the activity when the conventional TUG is performed, with a time 33% higher when compared with the mean values of the other subjects. In addition, the comparison between the different TUG approaches shows that the TUG combined with another task leads to a higher execution time considering the mean for all subjects, whereas the cognitive tasks present the longest mean time of execution. In addition, Figure 15c,d present the angles in the sagittal and frontal planes, respectively. In this analysis, there is an increase in the maximum angle when the TUG is combined with the cognitive task with mean sagittal angles of 40.0°, 46.4°, and 37.2° for the conventional TUG, TUG combined with cognitive tasks, and TUG combined with motor tasks, respectively. Similarly, the mean maximum angles at the frontal plane are 6.25° for the conventional TUG, 7.80° for the TUG combined with cognitive tasks, and 7.36° for the TUG combined with motor tasks.

The results and analysis enabled by the proposed low-cost sensor system demonstrate a fully integrated approach with important contributions to remote health monitoring and the accessibility of highly reliable gait analysis with protocol automatization to the general population, which can further help healthcare professionals in analysis and test implementation. The AI-enabled and centralized data transfer results in an accurate and user-friendly interface that can be operated by any professional or user. Considering only the production costs with the cost of the commercial components used in the optoelectronic sensor system, the total cost of the system is around USD 30 (around USD 5 for the ESP32; USD 15 for the IMU; USD 5 for the optical fiber system, including the light source and photodetectors; and USD 5 for batteries and peripheral components). The comparison between different approaches previously presented for the TUG test evaluation is presented in Table 5, where a comparison is made considering different sensors, namely only accelerometers, camera-based with markers, markerless cameras, pressure sensors, and optoelectronic sensor systems. The analysis is compared in terms of the detected transitions (i.e., the phases of the TUG tests), physiological monitoring, cost, portability (the possibility of remote monitoring), and detection principles.

## 4. Conclusions

This paper presented the development and application of an AI-assisted low-cost optoelectronic sensor system for biomechanical and physiological analysis. The sensor system is based on a low-cost IMU and an optical fiber-integrated chest belt for breathing rate monitoring, where all sensor systems have wireless connection with a gateway that sends the data to the cloud. The sensor system is characterized as a function of reference data and the RMSEs between the sensor responses are 0.6 BPM for the breathing rate and 0.037 m/s^2^ considering the mean of all axes for the IMU in the acceleration assessment. Similarly, for the angular velocity assessment, an RMSE of 0.27 °/s is obtained. Then, we use an unsupervised approach for the clustering of breathing rate signals, whereas the supervised approach is employed to obtain the spatiotemporal parameters of the balance and TUG tests. The balance and TUG tests performed with 12 subjects demonstrate the feasibility of the proposed device on biomechanical and physical therapy protocol automatization and assessment. The balance tests are performed in two different conditions, with a wider and narrower base, whereas the TUG tests are conducted with a combination of cognitive and motor tests. Therefore, the proposed approach results in a low-cost and fully automated sensor system that can be used in different protocols for physical rehabilitation. This study provides a feasible basis for large-scale clinical studies, as the combination of low-cost sensors, wireless communication, and AI-based analysis allows scalable deployment in aging societies. Future works include the use of the proposed device in remote health monitoring with the real-time analysis of patients in their daily activities at their homes.Another future work includes the implementation of the sensor system with different subject groups and a comparison of the results with gold-standard methods for gait analysis (marker-based camera systems) and for respiration analysis (respiratory belts).

## Figures and Tables

**Table 1:** Information about the subjects for the validation tests.

Subject	Gender	Age	Clinical Conditions	Mobility
1	Male	70 years	Hypertension Diabetes mellitus	Normal
2	Male	61 years	Hypertension Hearing impariment	Normal
3	Female	67 years	Epilepsy Asthma	Normal
4	Female	65 years	Hypertension Diabetes mellitus	Normal
5	Female	57 years	Hypertension	Normal
6	Male	75 years	Hypertension Diabetes mellitus	Normal
7	Female	70 years	Hypertension Diabetes mellitus	Normal
8	Female	71 years	Hypertension Diabetes mellitus	Normal
9	Female	53 years	Hypertension Diabetes mellitus	Normal
10	Female	67 years	Diabetes mellitus	Normal
11	Female	50 years	Diabetes mellitus	Normal
12	Female	57 years	Hypertension	Normal

**Table 2:** Comparison between the commercial and the proposed low-cost IMU for all parameters and axes.

Parameter	Axis	Error	Standard Deviation
Accelerometer	x-axis	0.060 m/s^2^	0.323 m/s^2^
Accelerometer	y-axis	0.010 m/s^2^	0.352 m/s^2^
Accelerometer	z-axis	0.042 m/s^2^	0.356 m/s^2^
Angular velocity	x-axis	0.25 °/s	7.13 °/s
Angular velocity	y-axis	0.24 °/s	8.18 °/s
Angular velocity	z-axis	0.33 °/s	4.48 °/s

**Table 3:** Biomechanical and physiological results of the sensor system during the different balance tests.

Test	Parameter	Mean Value
Wide base	Anterior displacement	33 mm
Wide base	Medial displacement	6 mm
Wide base	LFS	1.68
Narrow base	Anterior displacement	20 mm
Narrow base	Medial displacement	16 mm
Narrow base	LFS	0.92

**Table 4:** Spatiotemporal results of the sensor system during the different TUG tests.

Test	Total Time	Sagittal Angle	Frontal Angle
TUG	10.96 s	35°	5°
TUG+Motor	12.01 s	47°	8°
TUG+Cognitive	11.63 s	35°	5°

**Table 5:** Comparison between different sensing technologies for TUG automatic evaluation.

Principle	Detected Transitions	Physiological	Cost	Portability
Camera [37]	7	No	High	No
Markerless system [38]	7	No	Medium	No
Accelerometers [39]	5	No	Low	Yes
Pressure sensors [40]	6	No	Low	No
This paper	7	Respiration	Low	Yes

## Data Availability

The raw data supporting the conclusions of this article will be made available by the authors on request.
